# Retinal and choroidal angiogenesis: a review of new targets

**DOI:** 10.1186/s40942-017-0084-9

**Published:** 2017-08-21

**Authors:** Thiago Cabral, Luiz Guilherme M. Mello, Luiz H. Lima, Júlia Polido, Caio V. Regatieri, Rubens Belfort, Vinit B. Mahajan

**Affiliations:** 1 Edward S Harkness Eye Institute, Columbia University, New York, NY USA; 2 Department of Ophthalmology, Federal University of São Paulo, São Paulo, Brazil; 3 Department of Ophthalmology, Federal University of Espírito Santo, Vitória, Brazil; 4 Omics Laboratory, Department of Ophthalmology, Byers Eye Institute, Stanford University, Palo Alto, CA 94304 USA; 5 Department of Ophthalmology, Stanford University, Palo Alto, CA USA

**Keywords:** Age-related macular degeneration, Angiogenesis, Anti-angiogenesis, Choroidal neovascularization, Cytokine, Ocular neovascularization, Targets, Vascular endothelial growth factor

## Abstract

Retinal and choroidal neovascularization are a major cause of significant visual impairment, worldwide. Understanding the various factors involved in the accompanying physiopathology is vital for development of novel treatments, and most important, for preserving patient vision. The intraocular use of anti-vascular endothelial growth factor therapeutics has improved management of the retinal and choroidal neovascularization but some patients do not respond, suggesting other vascular mediators may also contribute to ocular angiogenesis. Several recent studies examined possible new targets for future anti-angiogenic therapies. Potential targets of retinal and choroidal neovascularization therapy include members of the platelet-derived growth factor family, vascular endothelial growth factor sub-family, epidermal growth factor family, fibroblast growth factor family, transforming growth factor-β superfamily (TGF-β1, activins, follistatin and bone morphogenetic proteins), angiopoietin-like family, galectins family, integrin superfamily, as well as pigment epithelium derived factor, hepatocyte growth factor, angiopoietins, endothelins, hypoxia-inducible factors, insulin-like growth factors, cytokines, matrix metalloproteinases and their inhibitors and glycosylation proteins. This review highlights current antiangiogenic therapies under development, and discusses future retinal and choroidal pro- and anti-angiogenic targets as wells as the importance of developing of new drugs.

**Electronic supplementary material:**

The online version of this article (doi:10.1186/s40942-017-0084-9) contains supplementary material, which is available to authorized users.

## Background

Angiogenesis is controlled by a dynamic equilibrium between proangiogenic and anti-angiogenic factors. Several circumstances, such as ischemia, hypoxia or inflammation, can influence the balance in favor of neovascularization [1]. Pathological ocular angiogenesis, particularly in the retina and choroid, should be carefully controlled as it may lead to significant visual impairment [2]. Diabetic retinopathy, neovascular age-related macular degeneration (AMD), retinopathy of prematurity, and retinal vessel occlusion are major causes of angiogenesis-related vision loss [3]. Understanding the factors underlying ocular angiogenesis will help us identify new therapeutic targets.

Vascular endothelial growth factor (VEGF) is considered the most critical regulator of ocular angiogenesis [1]. VEGF is related to induction of endothelial cell migration and proliferation after hypoxia [2]. Pegaptanib, an old medication used against ocular angiogenesis, is an anti-VEGF aptamer that competitively binds the VEGF-A165 isoform [4]. Currently, important anti-VEGF therapies include bevacizumab, ranibizumab, aflibercept, ziv-aflibercept and conbercept [5]. Bevacizumab and ranibizumab are immunoglobulin antibodies (monoclonal and fragment, respectively) that bind to all isoforms of VEGF-A, reducing free VEGF-A [4]. Aflibercept and ziv-aflibercept are recombinant fusion proteins that form a VEGF trap (binds to both sides of the VEGF-A and -B dimer), also binding to placental growth factor (PlGF) [4, 6]. Conbercept is a full human DNA sequence that binds to VEGF-A, -B and -C, and to PlGF. Different from aflibercept and ziv-aflibercept, conbercept contains the fourth binding domain of VEGFR-2, which enhances the association rate of VEGF to the receptor [5].

The main therapies used for ocular angiogenesis are anti-VEGF ligands. However, some patients may present worsening eye disease, regardless of aggressive treatment with anti-VEGF agents, suggesting other vascular mediators contribute to ocular angiogenesis. Alternative angiogenic factors and pathways are important causes of anti-VEGF therapy failure; and several mechanisms have been proposed to explain resistance to anti-angiogenesis drugs [7].

Research in our laboratory indicates that after intravitreal injection of anti-VEGF therapeutics into eyes with neovascular disease, levels of aqueous VEGF initially decrease and the anatomical and patients’ physiological parameters improve. Over time, as a compensatory mechanism, however, other angiogenic factor increase in the eye, making the eyes in part resistant to anti-VEGF therapy and maintaining pro-angiogenic stimulus [8]. Therefore, discovery of new targets may help identify new treatments and improve retinal and choroidal neovascularization management, and most importantly, save a patient’s vision.

## Methodology

A systematic review of retinal and choroidal angiogenesis was performed with a combination of selected keywords in PubMed for studies published up to May 2017. In this review, we focused on possible ocular targets and their connections. After matching specific keywords (“angiogenesis,” “biomarker,” “eye,” “neovascularization,” “ocular,” “target” and “treatment”), manuscripts written in English, Portuguese, French and Spanish were manually selected. Additional literature research was performed with the purpose of providing specific information of the molecules and recent advances in neovascularization therapy. Potential angiogenesis-related targets and therapies were selected and will be discussed.

**Fig. 1:**
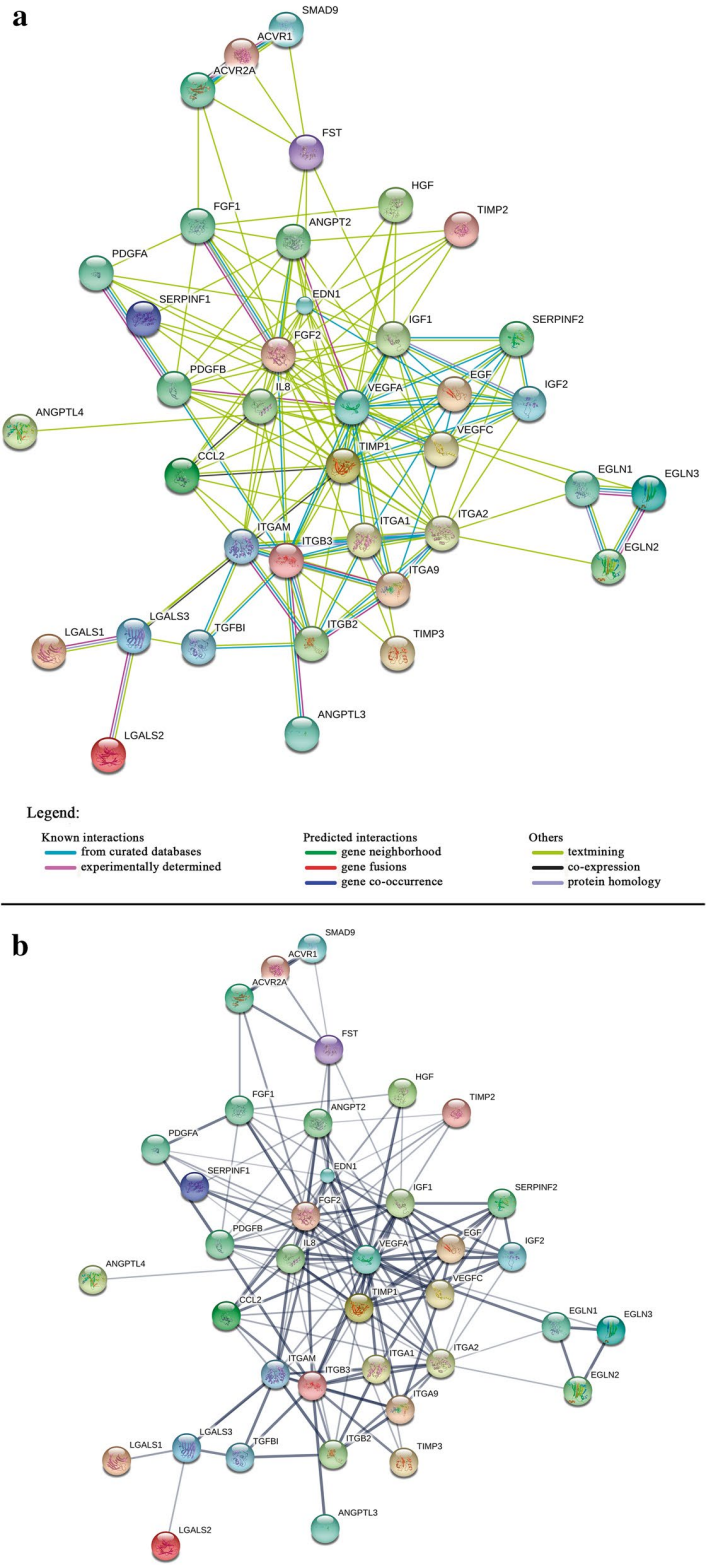
Protein–protein interaction (PPI) network of 38 potential angiogenesis-related proteins targets, based on STRING v10 data. This figure shows an important part of the retinal and choroidal angiogenesis-related PPI network and highlights the molecules with more and stronger interactions which we consider to be the main targets for future therapies. In this figure, proteins are represented as nodes, while interactions between them are represented as edges. Small and large nodes represent, respectively, proteins of unknown and known (or predicted) 3D structure. **a** *Colored lines* between the proteins indicate the various types of interaction evidence, as described in the figure legend. **b** Thickness indicates the strength of data support. Abbreviations: *ACVR1* activin A receptor, type I; *ACVR2A* activin A receptor, type IIA, *ANGPT2* angiopoietin 2, *ANGPTL3* angiopoietin-like 3, *ANGPTL4* angiopoietin-like 4, *EDN1* endothelin 1, *EGF* epidermal growth factor, *EGLN1* EGL nine homolog 1, *EGLN2* EGL nine homolog 2, *EGLN3* EGL nine homolog 3, *FGF1* fibroblast growth factor 1, *FGF2* fibroblast growth factor 2, *FST* follistatin, *HGF* hepatocyte growth factor, *IGF1* insulin-like growth factor 1 (somatomedin C), *IGF2* insulin-like growth factor 2 (somatomedin A), *IL8* interleukin 8, *CCL2* chemokine (C–C motif) ligand 2, *ITGA1* integrin, alpha 1, *ITGA2* integrin, alpha 2 (CD49B, alpha 2 subunit of VLA-2 receptor), *ITGAM* integrin, alpha M (complement component 3 receptor 3 subunit), *ITGB2* integrin, beta 2 (complement component 3 receptor 3 and 4 subunit), *ITGB3* integrin, beta 3 (platelet glycoprotein IIIa, antigen CD61), *ITGA9* integrin, alpha 9, *LGALS2* lectin, galactoside-binding, soluble, 2, *LGALS1* lectin, galactoside-binding, soluble, 1, *LGALS3* lectin, galactoside-binding, soluble, 3, *PDGFA* platelet-derived growth factor alpha polypeptide, *PDGFB* platelet-derived growth factor beta polypeptide, *SERPINF1* serpin peptidase inhibitor, clade F (alpha-2 antiplasmin, pigment epithelium derived factor), member 1, *SERPINF2* serpin peptidase inhibitor, clade F (alpha-2 antiplasmin, pigment epithelium derived factor), member 2, *SMAD9* SMAD family member 9, *TGFBI* transforming growth factor, beta-induced, *TIMP1* tissue inhibitor of metalloproteinases-1, *TIMP2* tissue inhibitor of metalloproteinases-2, *TIMP3* tissue inhibitor of metalloproteinases-3, *VEGFA* vascular endothelial growth factor A, *VEGFC* vascular endothelial growth factor C

To assess functional relevance of our candidates’ analyses were performed regarding binding partners, signaling pathways, transport molecules, and others. STRING (Search Tool for the Retrieval of Interacting Genes) is a large free online database of currently known proteins and their interactions, dedicated to functional associations between proteins. STRING analysis includes “interaction confidence scoring, comprehensive coverage (in terms of number of proteins, organisms and prediction methods), intuitive user interfaces and on a commitment to maintain a long-term, stable resource (since 2000)” [9]. A sequence of various sources are performed by the software to obtain the final protein networks: “(i) known experimental interactions are imported from primary databases, (ii) pathway knowledge is parsed from manually curated databases, (iii) automated text-mining is applied to uncover statistical and/or semantic links between proteins, based on Medline abstracts and a large collection of full-text articles, (iv) interactions are predicted de novo by a number of algorithms using genomic information as well as by co-expression analysis and (v) interactions that are observed in one organism are systematically transferred to other organisms, via pre-computed orthology relations” [9]. After a literature review we selected potential retinal and choroidal neovascularization targets, a protein–protein interaction network was retrieved from 42 proteins (organism searched: *Homo sapiens*), available at version 10.0 of STRING [9], at http://string-db.org/, with a minimum required interaction score of 0.400 (medium confidence). Four proteins (Angiopopietin-like-1 and -2, Tissue inhibitor of metalloproteinases-4 and Galectin-4) did not show interactions with the network and were removed to obtain the final scheme (Fig. 1) containing thirty-eight molecules (abbreviations used in the final search: ACVR1—activin A receptor, type I—, ACVR2A—activin A receptor, type II A—, ANGPT2—angiopoietin-like 2—, ANGPTL3—angiopoietin-like 3—, ANGPTL4—angiopoietin-like 4, CCL2—chemokine C–C motif ligand 2—, EDN1—endothelin 1—, EGF—epidermal growth factor—, EGLN1—egl nine homolog 1—, EGLN2—egl nine homolog 2—, EGLN3—egl nine homolog 3—, FGF1—fibroblast growth factor 1—, FGF2—fibroblast growth factor 2—, FST—follistatin—, HGF—hepatocyte growth fator—, IGF1—insulin-like growth factor 1—, IGF2—insulin-like growth factor 2—, IL8—interleukin 8—, ITGA1—integrin, alpha 1—, ITGA2—integrin, alpha 2—, ITGA9—integrin, alpha 9—, ITGAM—integrin, alpha M—, ITGB2—integrin, beta 2—, ITGB3—integrin, beta 3—, LGALS1—lectin, galactoside-binding, soluble, 1—, LGALS2—lectin, galactoside-binding, soluble, 2—, LGALS3—lectin, galactoside-binding, soluble, 3—, PDGFA—platelet-derived growth factor alpha polypeptide—, PDGFB—platelet-derived growth factor beta polypeptide—, SERPINF1—serpin peptidase inhibitor, clade F, member 1—, SERPINF2—serpin peptidase inhibitor, clade F, member 2—, SMAD9—SMAD family member 9—, TGFBI—transforming growth factor—, TIMP1—TIMP metallopeptidase inhibitor 1—, TIMP2—TIMP metallopeptidase inhibitor 2—, TIMP3—TIMP metallopeptidase inhibitor 3—, VEGFA—vascular endothelial growth factor A—and VEGFC—vascular endothelial growth factor C).

## Results

### Platelet-derived growth factor family

Platelet-derived growth factor (PDGF) is a mitogen, a chemoattractant for retinal pigment epithelium cells and retinal glia, and a critical factor for pericytes maintenance [10]. It is expressed as five isoforms (PDGF-AA, -AB, -BB, -CC and -DD) that can bind to different receptors (PDGFRα, PDGFRβ, and PDGFRα/β complexes) [11]. Although PDGF and VEGF families share a homology domain [11], and both participate in neovascular AMD pathogenesis, PDGF and VEGF levels appear to be inversely correlated [10]. Combined inhibition of PDGF and VEGF is reported to have stronger antiangiogenic effect than VEGF alone [12]. As PDGF exerts an important pro-angiogenic stimulus, the use of its antagonists has been widely investigated [13]. Indeed, maintaining the equilibrium between PDGF and VEGF may be a pivotal key for managing ocular neovascularization. Studies using PDGF inhibitors (such as Fovista and Axitinib), alone or in combination with other therapies, uncovered outcomes that might be useful for guiding management of ocular neovascularization [14, 15]. In vitro, Axitinib was shown to modulate VEGFR and PDGRF and inhibit endothelial cells angiogenesis [16]. However, recent reports found no benefits with adding Fovista and Rinucumab (an anti-PDGFRβ antibody) to anti-VEGF therapy in neovascular AMD, causing the scientific community to reassess the role of PDGF in ocular angiogenesis. [17]. In multicenter, randomized, double-masked, controlled phase III clinical trials for the treatment of wet age-related macular degeneration, OPH1002 and OPH1003, tested the combined therapy of anti-PDGF (1.5 mg of pegpleranib, Fovista^®^) with anti-VEGF (ranibizumab), versus Lucentis monotherapy, found no improvement in vision at 12 months [18].

To understand the possible sources of failure in these studies, it is important to consider recruitment and actions of pericytes. In new vessels, pericytes are controlled by many factors, including sphingosine-1-phosphate-1 (S1P-1), angiopoietins, and PDGF [19]. PDGF is critical to new vessel formation; and PDGF blockade dilatates capillaries [20]. Although pericytes will develop under conditions where the PDGF signaling pathway is disrupted, they are incapable of spreading along the newly formed vessels [21]. PDGF is required for growth and viability of pericytes, without which retinal neovascularization and capillary malformation worsen [19]. Thus, anti-PDGF drugs might allow us to control new blood vessel formation but not promote regression of existing ones. Further studies are necessary to better understand the impact of PDGF in the retinal and choroidal neovascularization.

### Vascular endothelial growth factor sub-family

The vascular endothelial growth factor sub-family, which includes five members (VEGF-A, -B, -C and -D, and PlGF), is the main target investigated in ocular angiogenesis, and part of platelet-derived growth factor family. VEGF-A is well established as an important factor contributing to neovascularization, and current anti-VEGF therapies focus on this form [7, 22]. However, other members of VEGF family also influence angiogenesis and have been recently investigated [5].

In 2006, Ikeda et al. [23] detected a markedly higher expression of VEGF-C and -D in the retinal pigment epithelium of AMD patients. Little is known about VEGF-D’s role in ocular angiogenesis, but VEGF-C’s importance has been increasingly investigated. Recently, Singh et al. [24] found that hypoxia-induced expression of VEGF-C in the retina is as potent as VEGF-A in inducing pathological retinal neovascularization. Our group suspects that VEGF-A blockade in isolation may engage a compensatory mechanism that increases the levels of other VEGF isoforms [8].

Pegaptanib, bevacizumab, ranibizumab, aflibercept, ziv-aflibercept and conbercept are important anti-VEGF drugs used to treat retinal and choroidal angiogenesis. Conbercept, a new and promising drug, was successfully reported to inhibit the three main VEGF isoforms (-A, -B and -C) and also PlGF [5], but more studies are needed to understand its impact in ocular angiogenesis management. Pegaptanib is an old anti-VEGF-A165 drug [4]. Bevacizumab, ranibizumab, aflibercept are currently the three main drugs used in ocular neovascularization therapy.

Cost-effectiveness is an issue that is of concern. The cost of these anti-VEGF therapeutics varies, limiting their use [25]. Ziv-aflibercept is a recombinant fusion protein that is reported to improve visual acuity in patients with neovascular AMD and to be a cheaper alternative to the same molecule aflibercept [26, 27]. The frequent need for repeated intravitreal anti-VEGF injections is an important factor affecting treatment costs. Development of a sustained-release delivery system against VEGF and other angiogenic factors may improve neovascularization management and its cost-effectiveness.

In recent years, the demand for new anti-angiogenic therapies has increased considerably. Various studies also reported no difference in VEGF levels in eyes with exudative AMD, suggesting the existence of alternative angiogenic pathways [28]. We believe that the anti-VEGF drugs, although still important, may not be the only player in future therapies of retinal and choroidal angiogenesis. Other VEGF-independent proteins and pathways are emerging for neovascular retinal diseases, so investigation into targeting other potential neovascularization-related proteins is essential.

### Pigment epithelium derived factor

Pigment epithelium derived factor (PEDF) is a member of the serine proteinase inhibitor (serpin) family with potent anti-angiogenic actions and protective effects against retina cell death [29]. Overexpression of PEDF molecules inhibits retinal and choroidal neovascularization [30]. PEDF can suppress angiogenic effects of hypoxia inducible factor-1 (HIF-1), decrease VEGF levels [31], and down-regulate MMP-2 and -9 expression and activities [32], promoting neovascularization regression. Equilibrium between PEDF and VEGF seems to be important for physiological retina development, as its imbalance may lead to pathological new vessels formation. A recent study showed that lentivirus-mediated PEDF gene transfer is effective treatment for choroidal neovascularization in an animal model [33]. More studies are needed to understand PEDF’s role as a therapeutic target of human ocular angiogenesis, as stimulation of PEDF pathway might offer a way to down-regulate angiogenic stimuli.

### Hepatocyte growth factor

Hepatocyte growth factor (HGF) is a potent cytokine that induces endothelial cell motility and growth, and contributes to blood vessels formation via c-MET signaling pathway [34]. HGF is also associated with migration of retinal pigment epithelium cells and disorganization of its intercellular junctions [35]. Studies suggest HGF is an important regulator of choroidal and retinal angiogenesis that is associated with proliferative diseases [36]. HGF has VEGF-independent functions in ocular angiogenesis and might be crucial to the initial phases of neovascularization [37]. It is possible that aqueous HGF levels increase after VEGF blockade [8], which would make HGF an important alternative target for controlling angiogenesis in the eye.

### Epidermal growth factor family

The epidermal growth factor (EGF) family consists of a large number of proteins with similarity to EGF in their biological activities and amino acid sequence. EGF and heparin-binding EGF-like growth factor (HB-EGF) are two similar members that seem to have important roles in ocular angiogenesis. HB-EGF is a potent stimulator of cell proliferation and migration that promotes angiogenesis in healing and recovery from injury [38]. Until now, it was not known how exactly HB-EGF functions within the retina and choroid, but it seems to be involved in some retinopathies [39]. Reduction of VEGF-A stimulates HB-EGF expression, which elicits an increase in VEGF-A levels in a positive feedback loop and contributes to angiogenesis [40]. HB-EGF may also induce a VEGF-independent angiogenesis [38]. Our group found that HB-EGF levels may increase after VEGF blockade [8]. Therefore, combined therapy against VEGF and HB-EGF pathways might promote longer and better control of retinal and choroidal neovascularization.

### Angiopoietins

The angiopoietins (ANG) are proteins that bind to Tie-2 (tyrosine kinase with immunoglobulin-like and EGF-like domains 2), regulating vascular development, maintenance and permeability [41]. Angiopoietin-1 (ANG-1) is a Tie-2 agonist that inhibits choroidal neovascularization formation and prevents vascular leakage [42]. Angiopoietin-2 (ANG-2), a Tie-2 antagonist interacts in a synergic mechanism with VEGF to promote destabilization of vessels, and regulates late stages of angiogenesis [43]. Curiously, a high ANG-2/VEGF ratio seems to promote vessels regression [44]. Therefore, equilibrium between ANG-1 and ANG-2, and their interaction with other angiogenic factors is essential for development and stabilization of new vessels.

Control of angiopoietins and their interactions with interaction may lead to better management of neovascular eye diseases. One study demonstrated that subretinal injections of adeno-associated virus-mediated gene therapy with cartilage oligomeric matrix protein angiopoietin-1 (AAV2.COMP-Ang1) reduced pro-angiogenic stimulus [45]. This highlights the importance of the angiopoietins in the ocular neovascularization process. Furthermore, recent studies demonstrated significant clinical advances in inhibiting the ocular ANG-2 angiogenic pathway, alone and in association with VEGF-A blockade [46]. CrossMAb is a promising bispecific domain-exchanged (crossed) monoclonal antibody that binds and neutralizes VEGF-A and ANG-2.

### Endothelins

Endothelin (ET) is expressed as three protein isoforms: endothelin-1 (predominant in ocular tissues and produced by endothelial cells and vascular smooth muscle cells), endothelin-2, and endothelin-3 [47]. ET-1 is considered a potent endogenous vasoconstrictor in small vessels, has an important regulatory role in retinal blood flow, and influences pericytes maintenance and replication [48]. Moreover, it may also induce angiogenesis and seems to be involved in advanced diabetic retinopathy pathogenesis [49]. Diabetes mellitus, insulin, glycaemia, hypoxia and oxidative stress are important stimuli that regulate endothelin levels [50]. In patients with type-2 diabetes, plasmatic ET-1 levels correlate with microangiopathy [51]. In recent publication, our group found that ET-1 levels increase after intravitreal injection of bevacizumab [8]. This may happen, at least in part, via the reduction of VEGF levels, as VEGF-A may have an inhibitory influence on ET-1 [52]. Nevertheless, due to ET-1’s role in diabetic microangiopathy and angiogenesis, it is reasonable to suppose that endothelins may be part of an alternative VEGF-induced angiogenesis pathway.

### Fibroblast growth factor family

Among several fibroblast growth factor (FGF) family members, FGF-1 and FGF-2 are two of the main angiogenesis-related factors [53]. FGF stimulates endothelial cell proliferation and migration by inducing proteases, integrins, and cadherin expression and consequent up-regulation of matrix metalloproteinases (MMP) and production of urokinase-type PA (activator of MMPs) [53]. The angiogenic effects of FGF-2 can occur in a VEGF-dependent or VEGF-independent manner and may be the first inducer of endothelial cell proliferation [54]. Furthermore, combined FGF-2 and VEGF blockade seems to be more effective against ocular angiogenesis than single VEGF blockade [55].

### Transforming growth factor-β superfamily: TGF-β1, activins, follistatin and bone morphogenetic proteins

*Transforming growth factor*-*β* (TGF-β) proteins, which consist of three types (-1, -2, and -3), have an important role in human disease. They stimulate extracellular matrix and cytokine production, and regulate endothelial cell proliferation and differentiation [56]. TGF-β1 promotes angiogenesis by up-regulation of NADPH oxidase 4 in endothelial cells [57]. It plays an important role in diabetic retinopathy [58]. However, until now few studies investigated the importance of TGF-β1 in ocular angiogenesis.

*Activin* is a distinct member of transforming growth factor-β superfamily, and produced by human retinal pigment epithelium [59]. It has also been reported to inhibit vascular endothelial cell proliferation [60] and to be involved in proliferative membrane pathogenesis [61]. *Activin* appears to act as an inhibitor of ocular angiogenesis, but more studies are needed to understand its role in the neovascularization process.

*Follistatin* (FST) is a regulator of *activin*, an important protein in hematopoiesis, the reproductive system, immunity, and especially inflammation [62]. Few studies have investigated the importance of FST in ocular angiogenesis and inflammatory diseases such as diabetes. *Follistatin* has been described as an inducer of angiogenesis and binding partner of angiogenin—an angiogenic protein synthesized in human choroid and retina and present at high levels in AMD eyes [63]. Although FST might be a VEGF-alternative route to angiogenesis [8], and activin an angiogenesis inhibitor factor, it is still uncertain how these proteins may influence retinal and choroidal neovascularization, and more studies are needed.

Another ocular angiogenesis-related factor from TGF-β superfamily is *bone morphogenetic protein* (BMP). BMP-9, also known as growth differentiation factor 2, is considered the most potent BMP family member to induce osteogenesis and a significant angiogenesis inhibitor [64]. BMP-9 can block VEGF and FGF-induced neovascularization [65]. Recently in mouse models, researchers showed activation of BMP-9 through the activin receptor-like kinase 1 endothelial receptor inhibits choroidal and oxygen-induced retinopathy neovascularization [66]. Over-expression of BMP-4 inhibits choroidal neovascularization in transgenic mice [67]. It is reasonable to believe that stimulation of a BMP pathway may be more powerful in reducing neovascularization than single VEGF inhibition, but more research is necessary to confirm this hypothesis.

### Angiopoietin-like family

The angiopoietin-like (ANGPTL) family may participate in several physiological and pathological processes, such as metabolism, hematopoiesis, inflammation and angiogenesis [68]. Among angiopoietin-like family proteins, ANGPTL-1, -2, -3 and -4 are proposed to have an important action in regulating angiogenesis [68]. In vitro, high glucose and hypoxia are important stimuli for retinal pigment epithelium cells, inducing angiogenic activity on human retinal endothelial cells, primarily mediated by ANGPL-4 [69, 70]; its angiogenic activity must be as potent as that of VEGF [70]. ANGPTL-4 was identified in areas of retina neovascularization, and its levels are increased in eyes with proliferative diabetic retinopathy [71–73]. Interestingly, ANGPTL-4 concentration seems to be independent of VEGF levels and increased by HIF-1, an important transcription factor linked to choroidal neovascularization [71, 72]. In addition, blockade of angiopoietin-like 4 with neutralizing antibodies reduces ocular angiogenesis [71]. Due to the influence of ANGPTL on systemic metabolism and in ocular angiogenesis, it may be an important systemic and ocular target for retinal and choroidal neovascularization, especially in diabetic patients.

### Galectins family and glycosylation process

Advanced glycation-end products are toxic lipids, proteins, and nucleic acids that become glycated after sugar exposure [74]. This is called glycosylation, a dynamic posttranslational process that creates binding sites for galectins. Glycan-binding proteins, also known as lectins, make up the galectin (Gal) family. Gal-1, -3, -8 and -9 can influence angiogenesis [75].

Gal-1 influences VEGF-receptor segregation, internalization, and trafficking, endothelial cell proliferation, migration and morphogenesis, and vascular permeability [75]. This molecule is also reported to preserve angiogenesis in anti-VEGF refractory tumors [76], which suggests galectins have ocular angiogenic stimulus independent of VEGF. Gal-1 is associated with diabetic retinopathy, and shown to co-localize with VEGF-receptor in neovascular eye tissues. Interestingly, in addition to its anti-VEGF effect, aflibercept, but not bevacizumab, neutralizes Gal-1 up-regulation of VEGF-receptor 2 phosphorylation [77]. Intravitreal injections of OTX008, a selective small-molecule inhibitor of Gal-1, was shown to reduce retinal neovascularization [78]. Gal-3 is a mediator of VEGF and FGF angiogenic stimuli, and like Gal-1, influences cell surface expression and activation of the VEGF-receptor [79]. Gal-3 also induces MMP expression and its related-angiogenic processes [80]. In a mouse model of diabetic retinopathy, retinal angiogenesis is regulated by advanced glycation-end product interactions with Gal-3 [81]. The functions of Gal-8 and -9 in the angiogenic process are still poorly understood. Recently, Lebecetin (a c-type lectin) was shown to inhibit retinal and choroidal neovascularization in an animal model [82]. Glycosylation and its related molecules are part of a wide area of intra- and extracellular pathways. Although galectins are related to the VEGF signaling pathway, they appear to exert contribute to new blood vessel formation and its blockade merits further study [78, 83].

### Hypoxia-inducible factors

Hypoxia-inducible factor (HIF-1), a DNA-binding transcription factor, is an important mediator of hypoxia-related angiogenesis. HIF-1 up-regulates multiple angiogenic genes including *VEGF* [84]. HIF-1 is associated with choroidal neovascularization [84] and seems to be a critical transcriptional factor in retinal angiogenesis [85]. Furthermore, therapy against HIF-1 markedly reduces choroidal, retinal, and retinal pigment epithelial angiogenesis [86]. Suppression of HIF may be a possible therapeutic strategy against ocular angiogenesis [87]. Specnuezhenide, a molecule of the fruit of *Ligustrum lucidum*, was shown to reduce retinal neovascularization by inhibiting the HIF-1/VEGF pathway [88]. Recently, Wert et al. [89] developed an animal model for proliferative diabetic retinopathy associated with elevated HIF-1 levels that may be helpful for unraveling HIF signaling pathways and testing antiangiogenic therapies.

### Insulin-like growth factors

Insulin-like growth factors (IGF) stimulate cell proliferation, differentiation, and neovascularization, regulated by IGF-binding proteins (IGFBP) [90]. IGFBP-4, -5 and -6 inhibit angiogenesis, while IGFBP-2 stimulates angiogenesis and has been founded at higher levels in patients with neovascular AMD [91, 92]. There are two types of IGF (-1 and -2). IGF-1 is associated with diabetic retinopathy, increased retinal vascular permeability, and retinal and choroidal neovascularization [93]. In transgenic mice expressing a GH antagonist, blockage of retina neovascularization was associated with down-regulation of IGF-1 levels, without significant alteration of VEGF-expression [94]. IGF-2 also stimulates angiogenesis, but it can act directly or in a VEGF-dependent manner, probably through MAPK pathway [90]. In addition, it was shown that IGF-I/IGF-II transgenic mice developed less choroidal neovascular membranes and fluorescein leakage than controls [95]. In conclusion, modulation of insulin-like growth factors may promote a better control of retinal and choroidal neovascularization beyond current available therapy.

### Cytokines

Chemokines are a family of small proteins classified into four different groups: CC, CXC, CX3C and C chemokines [96]. CXC group is composed by several important regulators of angiogenesis. The ELR-CXC chemokines [GRO-α (CXCL1), GRO-β (CXCL2), GRO-γ (CXCL3), ENA-78 (CXCL5), GCP-2 (CXCL6), NAP-2 (CXCL7) and IL-8 (CXCL8)] promote angiogenesis, while non-ELR ligands [CXCL4 (PF4), CXCL9 (Mig), CXCL10 (IP-10), I-TAC (CXCL11), SDF-1 (CXCL12) and BRAK (CXCL14)] appear to inhibit angiogenesis [97]. CC proteins, especially CCL2, have also been linked to ocular neovascularization [97]. A recent study demonstrated higher levels of chemokines in neovascular AMD eyes than controls and authors proposed that they might be potential angiogenesis targets [98].

IL-8 is a proinflammatory cytokine that is primarily involved in acute and chronic inflammatory processes. It is also related to ocular angiogenesis and increased vascular permeability [99]. Elevated IL-8 was founded in eyes with diabetic retinopathy [99]. Moreover, alterations of inflammatory signals, such as IL-8, must be involved in AMD pathogenesis and in the response to anti-VEGF therapy [100]. In patients with diabetic macular edema who are unresponsive to intravitreal bevacizumab, intravitreal triamcinolone plays an important role in decreasing central subfield thickness in association with IL-8 reduction [101]. In addition, combined therapy with intravitreal bevacizumab and triamcinolone has been effective for choroidal neovascularization unresponsive to anti-VEGF monotherapy [102]. This finding may be explained by the increased levels of IL-8 after VEGF blockade [8], as IL-8 might be an alternative angiogenesis pathway.

In eyes with choroidal neovascularization, elevated IL-17 was detected in aqueous humor when compared with the control group, leading to the hypothesis that this cytokine may stimulate angiogenesis in a VEGF-independent manner [103]. IL-10 is another interleukin that can promote pathological neovascularization [104]. Interleukins -4 [105], -12 [106] and -33 [107] may also inhibit ocular angiogenesis. Given this complexity, it may be difficult to understand and control every cytokine action and network, but management of neovascularization might only require control of a few, especially IL-8 and the CXC chemokines.

### Matrix metalloproteinases

Matrix metalloproteinases are enzymes that degrade extracellular matrix proteins. These proteases facilitate endothelial cell penetration in the sub-endothelial matrix, cell proliferation, and development of new vessels [108]. MMP-2 and -9 are involved in retinal and choroidal angiogenesis, and studies showed that inhibition of these MMPs can decrease ocular neovascularization [109]. In opposition to MMP, tissue inhibitors of metalloproteinases (TIMP-1, TIMP-2, TIMP-3 and TIMP-4) promote maintenance of extracellular matrix and are reported as inhibitors of angiogenesis. However, TIMP anti-angiogenic proprieties may be independent of MMP inhibition [110]. Regulating MMP and TIMP functions may be crucial for proper neovascularization therapy, but few studies have investigated how significant these molecules are during disease or their blockade for managing retinal and choroidal angiogenesis.

### Integrin superfamily

Integrins, transmembrane cell adhesion receptors that bind to extracellular matrix proteins, are involved in cell migration and angiogenesis. Their expression has been detected in neovascular ocular tissues [111]. In vitro and in animal models, integrin antagonism might be useful for treating neovascular ocular diseases [112, 113]. Combined integrin and VEGF blockade for ocular angiogenesis was reported to be more effective than anti-VEGF therapy alone [114]. Recently, the use of Tat PTD-Endostatin-RGD, via eye drops, bound integrin and blocked ocular neovascularization [115]. Despite progress, little is known about the role of integrins in ocular-related angiogenesis pathophysiology and whether integrin inhibition could be an effective option for inhibiting human retinal and choroidal angiogenesis.

## Conclusion

Pathological angiogenesis control is a difficult but necessary task for improving many patient’s visual prognosis and quality of life. The discovery of anti-VEGF drugs revolutionized the management of ocular neovascularization, but in many cases, single VEGF blockade is insufficient and additional anti-angiogenic targets and other strategies are required. Resistance to anti-VEGF therapy can be rooted in several causes: tolerance or tachyphylaxis to anti-VEGF drugs, alteration of the neovascular architecture, compensatory mechanisms, alternative angiogenic pathways, and genetic variations [7]. Previous studies have shown that changing the anti-VEGF therapeutic may be beneficial in exudative-AMD cases resistant to anti-VEGF drugs [116], although some important methodological flaws (e.g. absence of a comparison group) compromise the interpretation of the results and more robust studies are necessary [117]. However, considering that neovascularization is a dynamic process influenced by various regulatory mechanisms, the control of other pro- and anti-angiogenic pathways is the future of ocular angiogenesis therapy. To direct the efforts, it is necessary to understand the promising molecules and possible targets of angiogenic eye diseases. Moreover, to appropriately manage angiogenic diseases, future clinical studies must determine where these angiogenic factors exerts their main actions: retinal and/or choroidal vasculature. The new optical coherence tomography angiography will be a useful tool, in conjunction with other complementary tests, to better understand the response of retinal and choroidal vascular diseases (e.g., AMD, retinopathy of prematurity, diabetic macular edema, retinal vein occlusion) to different anti-angiogenic treatments [118]. All reported targets, their angiogenic influence, and current drugs reviewed in this study are summarized in Additional file 1.

A network of factors drives retinal and choroidal neovascularization physiopathology. Over the past years, several molecules were reported to play a pivotal role in ocular angiogenesis. Regulating different targets of this network appears to be more effective than focusing on any single one. We list the relevant retinal and choroidal factors that should be investigated further as targets of future anti-angiogenic therapies. After reviewing the current angiogenesis-related literature and select potential targets, a protein–protein interaction network of potential angiogenesis-related proteins was created using the STRING database (Fig. 1a, b). It is essential to understand which of these molecules might represent important therapeutic targets. Figure 1a indicates various types of interaction evidence (represented by edges colors), while Fig. 1b indicates the strength of data support (represented by edges thickness). There are thirty-eight nodes, one hundred fifty-three edges, with a clustering coefficient of 0.703 and a protein–protein interaction enrichment p value of 0. According to STRING software, this means that selected proteins “have more interactions among themselves than what would be expected for a random set of proteins of similar size, drawn from the genome. Such an enrichment indicates that the proteins are at least partially biologically connected as a group.” In extrapolating Fig. 1 data, it is reasonable to believe that the more interactions a protein has, the more important must be its role in angiogenesis regulation. In addition, the generated network highlights that the blockage of a single pro-angiogenic pathway may be insufficient to completely cease retinal and choroidal angiogenic stimulus.

Blockage or enhancement of different pathways and gene therapy are currently the most researched methods for facing neovascularization (Additional file 1). Alternative ocular drugs that are still under research and development include Conbercept (anti-VEGF-A, -B and -C and anti-PlGF) [5], CrossMAb (binds, neutralizes, and depletes VEGF-A and ANG-2) [46], Volociximab (integrin antagonists) [119], and many others [120]. Ongoing clinical trials evaluating new treatments against retinal and choroidal angiogenesis are summarized in Table 1. Research efforts should focus on key targets in the angiogenic pathway to accelerate discovery of new therapies and determine which are most effective. Beyond determining which molecules have the greatest impact on angiogenic stimuli, future studies need to set the importance of each one in retinal and choroidal diseases. The combination of blockades and/or enhancements of different molecules, to manage complex angiogenesis-related protein–protein interactions is probably the future of retinal and choroidal neovascularization treatment. This review summarizes and highlights current and future molecular targets that have a significant impact on the angiogenesis-related network that should direct future research.

**Table 1:** Ongoing clinical trials of new drugs against retinal and choroidal angiogenesis

NCT number	Drug	Target/mechanism	Phase	Conditions
NCT02543229	OPT-302	VEGF-C VEGF-D	1	Eye diseases, macular degeneration, retinal diseases, retinal degeneration, pathologic neovascularization
NCT02591914	E10030 (Fovista)	PDGF	1	Neovascular age-related macular degeneration
NCT01940887			3	
NCT02348359	X-82	VEGF	2	Age-related macular degeneration, macular degeneration, exudative age-related macular degeneration, age-related macular degeneration, eye diseases, retinal degeneration, retinal diseases
		PDGF		
NCT02699450	RO6867461	VEGF	2	Diabetic macular edema
		ANG-2		
NCT02530918	DS-7080ª	Robo-4	1	Neovascular age-related macular degeneration
NCT02727881	OHR-102 (Squalamine lactate)	VEGF	3	Age-related macular degeneration
		PDGF		
		bFGF		
NCT02857517	Conbercept	VEGF-A	2	Proliferative diabetic retinopathy, idiopathic choroidal neovascularization, retinal vein occlusion, polypoidal choroidal vasculopathy, branch retinal vein occlusion, macular edema, neovascular glaucoma, age-related macular degeneration, pathological myopia, diabetic macular edema, wet age-related macular degeneration
NCT02911311		VEGF-B	NP	
NCT03108352		VEGF-C	3	
NCT03159884		PlGF	4	
NCT03154892			NP	
NCT03128463			NP	
NCT02194634			3	
NCT03054818			NP	
NCT01024998	AAV2-sFLT01	Vector to neutralizes VEGF	1	Macular degeneration, age-related maculopathies, age-related maculopathy, retinal degeneration, retinal neovascularization, gene therapy, eye diseases
NCT02307682	RTH258 (Brolucizumab)	VEGF-A	3	Neovascular age-related macular degeneration
NCT02434328				
NCT02713204	REGN910 (Nesvacumab)	Inactivates the Tie2 receptor ligand ANG-2	2	Neovascular age-related macular degeneration
NCT02555306	DE-122	Endoglin	2	Age-related macular degeneration
NCT02914639	SF0166 Topical Ophthalmic Solution	Integrin αvβ3	2	Age-related macular degeneration
NCT02462928	Abicipar pegol	VEGF	3	Macular degeneration
		PDGF		
NCT02867735	LKA651	Erythropoietin	1	Macular edema, diabetic macular edema, neovascular age-related macular degeneration, retinal vein occlusions
NCT02613559	TK001 (Sevacizumab)	VEGF	1	Neovascular age-related macular degeneration
NCT02484690	RG7716	VEGF	2	Macular degeneration, choroidal neovascularization
		ANG-2		
NCT03066258	RGX-314 gene therapy	VEGF	1	Neovascular age-related macular degeneration, wet age-related macular degeneration

From http://www.clinicaltrials.gov. Accessed June 11, 2017. Searched terms: ocular angiogenesis, age-related macular degeneration, retinal neovascularization, choroidal neovascularization; Recruitment: “closed studies/*active, not recruiting*” AND “open studies*/recruiting*”. Abbreviations: ANG-2, angiopoietin-2; bFGF, Basic fibroblast growth factor; NCT, National Clinical Trial; PlGF, Placental growth factor; Robo-4, Roundabout Guidance Receptor-4; NP, not provided; VEGF, vascular endothelial growth factor

## Additional file

**Additional file 1.** Groups, subtypes, ocular angiogenesis influence and respectively drugs of ocular angiogenesis-related targets. A table containing information about groups, subtypes, ocular angiogenesis influence and, respectively, therapies for ocular angiogenesis-related targets.

## Abbreviations

AMD: age-related macular degeneration
ANG: angiopoietin
ANGPTL: angiopoietin-like
BMP: bone morphogenetic protein
EGF: epidermal growth factor
ET: endothelin
FGF: fibroblast growth factor
FST: follistatin
Gal: galectin
HB-EGF: heparin-binding EGF-like growth factor
HGF: hepatocyte growth factor
HIF: hypoxia inducible factor
IGF: insulin-like growth factors
IGFBP: IGF-binding protein
MMP: matrix metalloproteinases
PLGF: placental growth factor
PDGF: platelet-derived growth factor
PEDF: pigment epithelium derived factor
STRING: Search Tool for the Retrieval of Interacting Genes
TGF-ß: transforming growth factor-β
TIMP: tissue inhibitors of metalloproteinases
VEGF: vascular endothelial growth factor
