# Supplementary material for: Retinal and choroidal angiogenesis: a review of new targets
Source: Int J Retina Vitreous. 2017 Aug 21;3:31. doi: 10.1186/s40942-017-0084-9 (PMC5563895; doi:10.1186/s40942-017-0084-9)
Supplement: Supplementary file 1 — Additional file 1. Groups, subtypes, ocular angiogenesis influence and respectively drugs of ocular angiogenesis-related targets. A table containing information about groups, subtypes, ocular angiogenesis influence and, respectively, therapies for ocular angiogenesis-related targets. [file 40942_2017_84_MOESM1_ESM.docx]

**Additional file 1**. Groups, subtypes, ocular angiogenesis influence and respectively drugs of ocular angiogenesis-related targets

| Groups | | | Subtypes | Ocular Angiogenesis | Drugs |
| --- | --- | --- | --- | --- | --- |
| PDGF family | | | PDGF (-AA, -AB, -BB, -CC, -DD) | Pro | Fovista  CR002 (against PDGF-D)  HL-217 |
|  | | | PDGF receptor |  | Axitinib  GW771806  Vatalanib  Rinucumab |
| VEGF sub-family | | | VEGF-A | Pro | Aflibercept  Bevacizumab  Conbercept  Pegaptanib (VEGF-A165 isoform)  Ranibizumab  VF-Trap  rAAV.sFLT-1 |
|  |  |  | VEGF-B | Pro | Aflibercept  Conbercept  rAAV.sFLT-1 |
|  |  |  | VEGF-C | Pro | Conbercept  KML001 |
|  |  |  | VEGF receptor |  | Vatalanib |
|  |  |  | PlGF | Pro | Aflibercept  Conbercept  rAAV.sFLT-1 |
| PEDF | | | PEDF | Anti | AAV2-hPEDF |
| HGF | | | HGF | Pro |  |
| EGF family | | | HB-EGF | Pro | Cetuximab ^a^ |
| Angiopoietins | | | Angiopoietin-1 | Anti | AAV2.COMP-Ang1  AMG-386 |
|  |  |  | Angiopoietin-2 | Pro | CrossMAb  AMG-386 |
| Endothelins | | | Endothelin-1 | Pro |  |
| FGF family | | | FGF-1 | Pro |  |
|  |  |  | FGF-2 | Pro | VF-Trap |
|  | | | FGF receptor |  |  |
| TFG-ß superfamily | | | TFG-ß1 | Pro |  |
|  |  |  | Activins | Anti |  |
|  |  |  | Follistatin | Pro |  |
|  |  |  | BMP-4 | Anti |  |
|  |  |  | BMP-9 | Anti | BMP-9 adenoviral particle |
| Angiopoietin-like family | | | ANGPTL-1 | Pro/Anti |  |
|  |  |  | ANGPTL-2 | Pro |  |
|  |  |  | ANGPTL-3 | Pro |  |
|  |  |  | ANGPTL-4 | Pro | ANGPTL4 neutralizing antibody |
|  |  |  | ANGPTL-6 | Pro |  |
| Galectins family and glycosylation process | | | Gal-1 | Pro | Aflibercept  OTX008 |
|  |  |  | Gal-3 | Pro |  |
| HIF | | | HIF-1 | Pro | Specnuezhenide  siRNAs for HIF-1α and -2α |
| IGF | | | IGF-1 | Pro |  |
|  |  |  | IGF-2 | Pro | DX-2647 |
|  |  |  | IGFBP-2 | Pro |  |
|  |  |  | IGFBP-4 | Anti |  |
|  |  |  | IGFBP-5 | Anti |  |
|  |  |  | IGFBP-6 | Anti |  |
| Cytokines | |  |  |  |  |
|  | ELR-CXC chemokines | | GRO-α (CXCL1) | Pro |  |
|  |  |  | GRO-β (CXCL2) | Pro |  |
|  |  |  | GRO-γ (CXCL3) | Pro |  |
|  |  |  | ENA-78 (CXCL5) | Pro |  |
|  |  |  | GCP-2 (CXCL6) | Pro |  |
|  |  |  | NAP-2 (CXCL7) | Pro |  |
|  |  |  | IL-8 (CXCL8) | Pro | Triamcinolone  ABX-IL8 ^a^  SCH-479833 ^a^  SCH-527123 ^a^ |
|  | non-ELR-CXC chemokines | | PF4 (CXCL4) | Anti |  |
|  |  |  | MIG (CXCL9) | Anti |  |
|  |  |  | IP-10 (CXCL10) | Anti | Plasmid DNA-encoding CXCL10 ^a^ |
|  |  |  | I-TAC (CXCL11) | Anti |  |
|  |  |  | SDF-1 (CXCL12) | Anti |  |
|  |  |  | BRAK (CXCL14) | Anti |  |
|  | CC | | CCL2 | Pro |  |
| MMP | | | MMP-2 | Pro | AG3340  DPC-A37668 |
|  |  |  | MMP-9 | Pro | AG3340 |
|  |  |  | TIMP-1 | Anti |  |
|  |  |  | TIMP-2 | Anti |  |
|  |  |  | TIMP-3 | Anti | pEBAct-TIMP3 |
|  |  |  | TIMP-4 | Anti |  |
| Integrin superfamily | | | Integrins | Pro | ATN-161  EMD478761  JNJ-26076713  JSM6427  Tetraiodothyroacetic acid  Lebecetin |

^a^ Studies of cancer-related angiogenesis
